# Supplementary material for: Class III Alcohol Dehydrogenase Plays a Key Role in the Onset of Alcohol-Related/-Associated Liver Disease as an S-Nitrosoglutathione Reductase in Mice
Source: Int J Mol Sci. 2023 Jul 28;24(15):12102. doi: 10.3390/ijms241512102 (PMC10419236; doi:10.3390/ijms241512102)
Supplement: Supplementary file 1 [file ijms-24-12102-s001.zip › ijms-2460882-supplementary.pdf]

**Data Availability Statement**		(Raw data)		
**Fig. 2b Ratio of liver weight/body weight after 12 months of CAC**				
	mouse No.	Body weigt (g)	Liver weight (g)	LW/BW
WT(C)	1	48.1	1.678	0.034885655
	2	41.5	1.38	0.033253012
	3	48.4	2.188	0.045206612
	4	30.9	1.075	0.034789644
	5	44.2	1.42	0.032126697
	mean	42.62	1.5482	0.036325669
	SD	7.151013914	0.416784357	0.005244119
WT(E)	1	42.4	2.444	0.057641509
	2	43	1.915	0.044534884
	3	42.9	2.4	0.055944056
	4	41	2.214	0.054
	5	42.6	2.79	0.065492958
	mean	42.38	2.3526	0.055512034
	SD	0.80746517	0.321178766	0.007535311
Adh3-/-(C	1	40.3	2.49	0.0617866
	2	39.9	1.55	0.038847118
	3	43	2.79	0.064883721
	4	32.3	1.53	0.047368421
	5	42.6	2.5	0.058685446
	mean	39.62	2.172	0.054820798
	SD	4.313583197	0.589423447	0.010887307
Adh3-/-(E	1	37.9	2.88	0.075989446
	2	36	2.24	0.062222222
	3	32.8	1.659	0.050579268
	4	31	1.336	0.043096774
	5	31.5	1.808	0.057396825
	mean	33.84	1.9846	0.058646572
	SD	2.990484911	0.596836494	0.012438671
Student t test (one-sided test)			*P*	
	**WT(E) vs WT(C)**		**0.000729782**	

Fig. 3b The rate of ORO staining positive area in liver

Group	Mouse No.	Photograph in the same preparation	% of positive area	Mean % of positive area in a mouse	Mean % of positive area in a group	SD
WT(C)	1	a	23.8	21.1	16.1	4.371
		b	18.1			
		c	23.3			
		d	19.0			
	2	a	11.3	13.0		
		b	16.4			
		c	15.0			
		d	9.3			
	3	a	13.0	14.2		
		b	15.9			
		c	20.6			
		d	7.4			
WT(E)	1	a	52.9	56.5	47.6	11.178
		b	61.4			
		c	56.5			
		d	55.1			
	2	a	35.8	35.1		
		b	34.3			
		c	34.8			
		d	35.4			
	3	a	58.9	51.4		
		b	40.4			
		c	55.7			
		d	50.5			
Adh3-/- (C)	1	a	3.1	1.3	8.0	8.295
		b	1.8			
		c	0.3			
		d	0.1			
	2	a	9.0	5.5		
		b	8.6			
		c	2.7			
		d	1.8			
	3	a	19.4	17.3		
		b	10.6			
		c	21.6			
		d	17.6			
Adh3-/- (E)	1	a	8.4	8.8	12.6	3.329
		b	11.7			
		c	9.8			
		d	5.5			
	2	a	14.7	15.0		
		b	8.6			
		c	22.4			
		d	14.4			
	3	a	9.8	14.0		
		b	10.6			
		c	18.3			
		d	17.4			

Student t test (one side test)	*P* *n=3 mice*
**WT(E) vs WT(C)**	**0.00517684**
**WT(E) vs *Adh3-/-*(E)**	**0.003245182**

**Fig. 3c Triglyceride (TG) content in the liver after 12 months of CAC**			
	mouse No.	TG (mg)/g liver	
WT(C)	1	5.076	
	2	8.786	
	3	6.032	
	4	7.878	
	5	4.789	
	mean	6.5122	
	SD	1.753443526	
WT(E)	1	11.07	
	2	9.543	
	3	13.799	
	4	13.321	
	5	15.865	
	mean	12.7196	
	SD	2.461315461	
Adh3-/- ((	1	4.934	
	2	7.143	
	3	7.89	
	4	5.012	
	5	4.356	
	mean	5.867	
	SD	1.549624148	
Adh3-/- (I	1	8.467	
	2	4.844	
	3	8.457	
	4	7.067	
	5	9.445	
	mean	7.656	
	SD	1.785644702	
Student t	test (one-sided test)		*P*
	**WT(E) vs WT(C)**		**0.00265739**
	**WT(E) vs *Adh3-/-* (E)**		**0.008762749**
	Adh3-/- (E) vs *Adh3-/-* (C)		0.193665374

Fig. 4 Serum AST and ALT of mice after 12 month CAC

	mouse No.	AST(IU/L)	ALT(IU/L)
WT(C)	1	54	30
	2	39	18
	3	59	50
	4	82	16
	5	106	31
	mean	68	29
	SD	26	14
WT(E)	1	200	75
	2	192	391
	3	236	171
	4	140	276
	5	82	115
	mean	170	206
	SD	60	128
Adh3-/- (	1	45	36
	2	60	55
	3	114	162
	4	188	148
	5	129	164
	mean	107	113
	SD	57	62
Adh3-/- (	1	61	44
	2	54	53
	3	47	50
	4	77	42
	5	85	108
	mean	65	59
	SD	15.8492902	27.52816739

Student t test(one-sided test)	AST	ALT
	P	*P*
**WT(E) vs WT(C)**	**0.0274379753109604**	**0.007767607**
**WT(E) vs *Adh3-/-* (E)**	**0.00265**	**0.018694465**
WT(E) vs *Adh3-/-* (C)	0.239180882212304	0.092243922
Adh3-/-(E) vs *Adh3-/-* (C)	0.074805162	0.058230186

Fig.6a ADH3 (GSNOR) mRNA in the liver

			GSNOR mRNA/		
	Month	mouse No	β actin mRNA	mean	SD
WT(C)	1	1	0.937	1.0004	0.109413436
WT(C)	1	2	0.875		
WT(C)	1	3	1.104		
WT(C)	1	4	0.96		
WT(C)	1	5	1.126		
WT(C)	4	1	0.742	0.6848	0.113996491
WT(C)	4	2	0.66		
WT(C)	4	3	0.706		
WT(C)	4	4	0.81		
WT(C)	4	5	0.506		
WT(C)	12	1	0.683	0.7652	0.136074244
WT(C)	12	2	0.926		
WT(C)	12	3	0.855		
WT(C)	12	4	0.583		
WT(C)	12	5	0.779		
WT(E)	1	1		1.145	0.234034898
WT(E)	1	2	1.396		
WT(E)	1	3	1.105		
WT(E)	1	4			
WT(E)	1	5	0.933		
WT(E)	4	1	0.933	0.949	0.142107354
WT(E)	4	2	1.171		
WT(E)	4	3	0.948		
WT(E)	4	4	0.775		
WT(E)	4	5	0.918		
WT(E)	12	1	1.39	1.01575	0.261768823
WT(E)	12	2	0.836		
WT(E)	12	3	1.003		
WT(E)	12	4			
WT(E)	12	5	0.834		
				P	
	**2 way ANOVA**	**WT(E) vs WT(C)**		**0.002**	
	**FTEST**	**WT(E) vs WT(C)**		**4.38504E-10**	
	Student t test (1M)	WT(E) vs WT(C)		0.134198523	
	**Student t test (4M)**	**WT(E) vs WT(C)**		**0.005914223**	
	**Student t test (12M)**	**WT(E)- vs WT(C)**		**0.051937016**	
	**Student t test (1-12M)**	**WT(E) vs WT(C)**		**0.005442316**	

Fig.6b GSNOR activity of the liver

	Month	mouse No.	GSNOR activity (unit)/ mg liver protein	mean	SD
WT(C)	1	1	9.213	7.0954	2.83989072
WT(C)	1	2	4.199		
WT(C)	1	3	4.62		
WT(C)	1	4	10.718		
WT(C)	1	5	6.727		
WT(C)	4	1	3.76	4.1528	1.273741222
WT(C)	4	2	4.135		
WT(C)	4	3	6.1		
WT(C)	4	4	4.21		
WT(C)	4	5	2.559		
WT(C)	12	1	0.942	1.8266	0.908625776
WT(C)	12	2	1.016		
WT(C)	12	3	3.167		
WT(C)	12	4	1.964		
WT(C)	12	5	2.044		
WT(E)	1	1	7.826	8.578	1.331305462
WT(E)	1	2	10.792		
WT(E)	1	3	8.649		
WT(E)	1	4	7.342		
WT(E)	1	5	8.2789		
WT(E)	4	1	5.198	6.6846	3.921566562
WT(E)	4	2	13.262		
WT(E)	4	3	5.001		
WT(E)	4	4	3.057		
WT(E)	4	5	6.905		
WT(E)	12	1	4.552	2.5442	1.411212316
WT(E)	12	2	1.805		
WT(E)	12	3	1.61		
WT(E)	12	4	1.268		
WT(E)	12	5	3.486		
			P		
	2 way ANOVA	WT(E) vs WT(C)	0.0639		
	**FTEST (1-12M)**	**WT(E) vs WT(C)**	**0.019335645**		
	Student t test (1M)	WT(E) vs WT(C)	0.160758111		
	Student t test (4M)	WT(E) vs WT(C)	0.103498887		
	Student t test (12M)	WT(E) vs WT(C)	0.183528909		
	Student t test (1-12M)	WT(E) vs WT(C)	0.092762396		

Fig. 2e Triglyceride (TG) content in the liver after 12 months of CAC			
	mouse No.	TG (mg)/g liver	
WT(C)	1	5.076	
	2	8.786	
	3	6.032	
	4	7.878	
	5	4.789	
	mean	6.5122	
	SD	1.753443526	
WT(E)	1	11.07	
	2	9.543	
	3	13.799	
	4	13.321	
	5	15.865	
	mean	12.7196	
	SD	2.461315461	
Adh3-/- ((	1	4.934	
	2	7.143	
	3	7.89	
	4	5.012	
	5	4.356	
	mean	5.867	
	SD	1.549624148	
Adh3-/- (I	1	8.467	
	2	4.844	
	3	8.457	
	4	7.067	
	5	9.445	
	mean	7.656	
	SD	1.785644702	
Student t	test (one-sided test)		*P*
	**WT(E) vs WT(C)**		**0.00265739**
	**WT(E) vs *Adh3-/-* (E)**		**0.008762749**
	Adh3-/- (E) vs *Adh3-/-* (C)		0.193665374

Fig. 3 Serum AST and ALT of mice after 12 month CAC

	mouse No.	AST(IU/L)	ALT(IU/L)
WT(C)	1	54	30
	2	39	18
	3	59	50
	4	82	16
	5	106	31
	mean	68	29
	SD	26	14
WT(E)	1	200	75
	2	192	391
	3	236	171
	4	140	276
	5	82	115
	mean	170	206
	SD	60	128
Adh3-/- (	1	45	36
	2	60	55
	3	114	162
	4	188	148
	5	129	164
	mean	107	113
	SD	57	62
Adh3-/- (	1	61	44
	2	54	53
	3	47	50
	4	77	42
	5	85	108
	mean	65	59
	SD	15.8492902	27.52816739

Student t test(one-sided test)	AST	ALT
	P	*P*
**WT(E) vs WT(C)**	**0.0274379753109604**	**0.007767607**
**WT(E) vs *Adh3-/-* (E)**	**0.00265**	**0.018694465**
WT(E) vs *Adh3-/-* (C)	0.239180882212304	0.092243922
Adh3-/-(E) vs *Adh3-/-* (C)	0.074805162	0.058230186

**Fig. 4b ADH3 (GSNOR) mRNA in the liver**					
			GSNOR mRNA/		
	Month	mouse No	β actin mRNA	mean	SD
WT(C)	1	1	0.937	1.0004	0.109413436
WT(C)	1	2	0.875		
WT(C)	1	3	1.104		
WT(C)	1	4	0.96		
WT(C)	1	5	1.126		
WT(C)	4	1	0.742	0.6848	0.113996491
WT(C)	4	2	0.66		
WT(C)	4	3	0.706		
WT(C)	4	4	0.81		
WT(C)	4	5	0.506		
WT(C)	12	1	0.683	0.7652	0.136074244
WT(C)	12	2	0.926		
WT(C)	12	3	0.855		
WT(C)	12	4	0.583		
WT(C)	12	5	0.779		
WT(E)	1	1		1.145	0.234034898
WT(E)	1	2	1.396		
WT(E)	1	3	1.105		
WT(E)	1	4			
WT(E)	1	5	0.933		
WT(E)	4	1	0.933	0.949	0.142107354
WT(E)	4	2	1.171		
WT(E)	4	3	0.948		
WT(E)	4	4	0.775		
WT(E)	4	5	0.918		
WT(E)	12	1	1.39	1.01575	0.261768823
WT(E)	12	2	0.836		
WT(E)	12	3	1.003		
WT(E)	12	4			
WT(E)	12	5	0.834		
				P	
	**2 way ANOVA**	**WT(E) vs WT(C)**		**0.002**	
	**FTEST**	**WT(E) vs WT(C)**		**4.38504E-10**	
	Student t test (1M)	WT(E) vs WT(C)		0.134198523	
	**Student t test (4M)**	**WT(E) vs WT(C)**		**0.005914223**	
	**Student t test (12M)**	**WT(E)- vs WT(C)**		**0.051937016**	
	**Student t test (1-12M)**	**WT(E) vs WT(C)**		**0.005442316**	

Fig. 4 Supplement GSNOR activity of the liver

			GSNOR activity (unit)/		
	Month	mouse No.	mg liver protein	mean	SD
WT(C)	1	1	9.213	7.0954	2.83989072
WT(C)	1	2	4.199		
WT(C)	1	3	4.62		
WT(C)	1	4	10.718		
WT(C)	1	5	6.727		
WT(C)	4	1	3.76	4.1528	1.273741222
WT(C)	4	2	4.135		
WT(C)	4	3	6.1		
WT(C)	4	4	4.21		
WT(C)	4	5	2.559		
WT(C)	12	1	0.942	1.8266	0.908625776
WT(C)	12	2	1.016		
WT(C)	12	3	3.167		
WT(C)	12	4	1.964		
WT(C)	12	5	2.044		
WT(E)	1	1	7.826	8.578	1.331305462
WT(E)	1	2	10.792		
WT(E)	1	3	8.649		
WT(E)	1	4	7.342		
WT(E)	1	5	8.2789		
WT(E)	4	1	5.198	6.6846	3.921566562
WT(E)	4	2	13.262		
WT(E)	4	3	5.001		
WT(E)	4	4	3.057		
WT(E)	4	5	6.905		
WT(E)	12	1	4.552	2.5442	1.411212316
WT(E)	12	2	1.805		
WT(E)	12	3	1.61		
WT(E)	12	4	1.268		
WT(E)	12	5	3.486		
			P		
	2 way ANOVA	WT(E) vs WT(C)	0.0639		
	**FTEST (1-12M)**	**WT(E) vs WT(C)**	**0.019335645**		
	Student t test (1M)	WT(E) vs WT(C)	0.160758111		
	Student t test (4M)	WT(E) vs WT(C)	0.103498887		
	Student t test (12M)	WT(E) vs WT(C)	0.183528909		
	Student t test (1-12M)	WT(E) vs WT(C)	0.092762396		
